# Protozoan Co‐Infection Drives Fish Mortality Event in Crete's Karteros River

**DOI:** 10.1111/jfd.14099

**Published:** 2025-02-24

**Authors:** Pantelis Katharios, Evangelia Karageorgou

**Affiliations:** ^1^ Institute of Marine Biology, Biotechnology & Aquaculture Hellenic Centre for Marine Research Heraklion Greece; ^2^ Department of Biology, School of Sciences and Engineering University of Crete Heraklion Greece

**Keywords:** fishkill, gill parasites, histopathology, Mugilidae

## Abstract

In August 2024, a significant fish kill involving hundreds of flathead grey mullet ( *Mugil cephalus* ) was reported in the delta region of the Karteros River, Crete, Greece. The investigation identified the primary cause of mortality as severe parasitic infections, specifically from the protozoan parasites *Amyloodinium ocellatum* and *Trichodina* sp., both of which heavily affected the gills of the fish. Concurrently, poor water quality in the area, likely due to reduced water volume and limited water renewal, created favourable conditions for parasite proliferation while weakening the fish's natural defences. This event raised concerns due to the location of the fish kill in the Karteros River delta, a highly protected area designated as a small island wetland.

## 1 Introduction

A fish kill is a significant and sudden event characterised by the large‐scale mortality of fish in a particular water body, that may involve both wild and cultured fish populations. The causes of such an event can be diverse and complex, often involving multiple contributing factors. These may include anthropogenic activities, such as pollution or habitat destruction, adverse environmental conditions like temperature fluctuations or oxygen depletion, and the outbreak of diseases within the fish population (La and Cooke 2011). In many instances, fish kills result from a combination of these factors, where environmental stressors weaken the fish, making them more susceptible to infections or other detrimental impacts. Understanding the specific causes behind a fish kill is crucial for mitigating future occurrences and protecting aquatic ecosystems.

Over the past decade, at least five fish kills have been documented in the water bodies of the Prefecture of Heraklion, Crete, Greece, all of which our research team was actively involved in investigating. The first incidence concerned a mass mortality of goldfish at the Aposelemis Dam, the primary source of potable water for northeast Crete. This mortality was attributed to heavy parasitism of the goldfish by the flagellate parasite *Ichthyobodo* sp. (Katharios et al. 2023). Three additional incidents occurred in subsequent years at the Aposelemis delta, involving mass mortality of flathead grey mullet ( *Mugil cephalus* ). These events were attributed to oxygen depletion resulting from eutrophication, which was caused by point source pollution of the Aposelemis River (Panthelis Katharios, unpublished data).

Here, we present our findings on the most recent mortality event recorded in the Karteros River, Heraklion, Crete, which also affected flathead grey mullets. The event occurred in early August 2024 and, like previous incidents, has raised concerns within the local community.

## 2 Materials and Methods

The fish mortality was reported on August 3, 2024, at the delta of the Karteros River (35°19′59″ N, 25°11′31″ E). Due to reduced water flow, the river's delta was no longer connected to the sea, resulting in the formation of a shallow pond composed mostly of stagnant water (File S1). The water displayed clear signs of eutrophication, including increased algae growth and decreased oxygen levels. Hundreds of fish lay dead along the riverbanks, while moribund fish with white lesions on their skin swam sluggishly near the water's surface.

### 2.1 Sampling

Both freshly dead and moribund fish were sampled (*n* = 15) and placed in an isothermal cooler for immediate transfer to the Aquaculture Microbiology lab at Institute of Marine Biology, Biotechnology and Aquaculture of the Hellenic Centre for Marine Research for examination. Water samples were collected in clean sampling bottles, with temperature and oxygen levels measured on‐site using a portable instrument (Handy Polaris 2, OxyGuard International A/S, Farum, Denmark). The water samples were then sent to a private accredited laboratory for analysis. The water was tested for a range of parameters, including colour, pH, conductivity, total hardness, alkalinity, chlorides, sulphates, fluorides, phosphates, nitrates, nitrites, ammoniates, BOD, COD, total suspended solids, phenols, anionic surfactants, fats & grease, as well as microbiological analysis for total coliforms and *E. coli* .

### 2.2 Gross Examination

Wet mounts from gill samples and skin scrapings were examined under a compound light microscope (Nikon Eclipse 50i). Photographs were captured using a Nikon microscope camera (DS‐Fi2). Parasite morphometrics were measured with ImageJ software, utilising microphotographs of formalin‐fixed specimens in wet mounts (*n* = 30).

### 2.3 Histology

Gill samples fixed with phosphate buffered formalin (10%) were progressively dehydrated in higher concentrations of ethanol (from 70% to 96% EtOH), embedded in glycol methacrylate resin (Technovit 7100, Heraeus Kulzer) and cut in 4 μm sections with a microtome (RM 2245, Leica, Germany). Sections were mounted on slides and stained with methylene blue/azure II/basic fuchsin (polychrome stain) (Bennett et al. 1976).

## 3 Results

Affected fish were swimming lethargically near the riverbanks and displayed the characteristic cloudy appearance of skin commonly associated with *Amyloodinium* infections. Several individuals, typically the largest ones, exhibited haemorrhages on their body trunks, primarily along their sides. Interestingly, mucous content in both skin and gills was not excessive.

**FIGURE 1:**
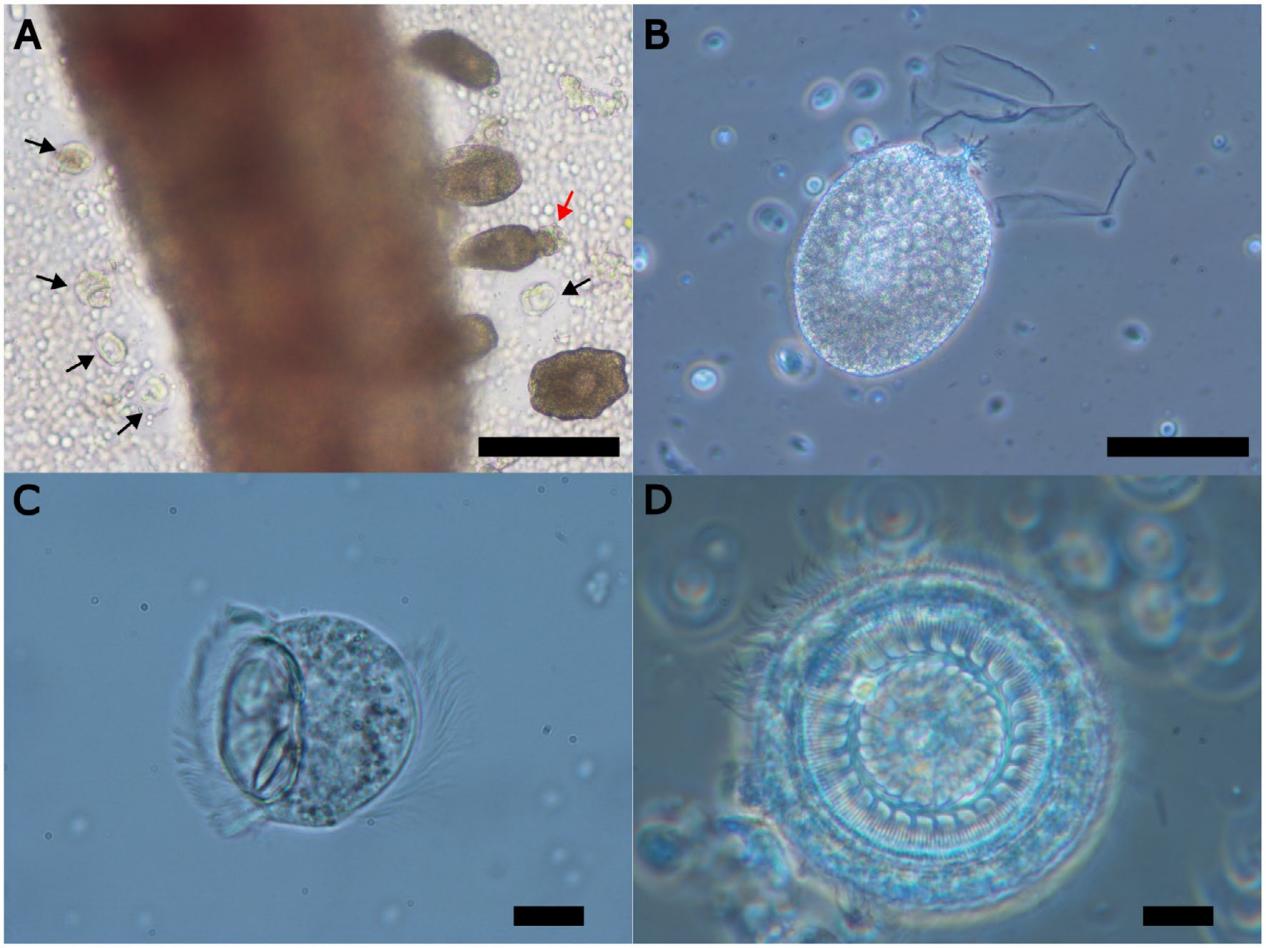
(A) Wet mount of grey mullet gill coinfected by *Amyloodinium ocellatum* and *Trichodina* sp. (black arrows). Red arrow points to a *Trichodina* sp. attached to an *Amyloodinium* trophont. Bar: 100 μm. (B) Phase contrast microphotograph of a detached *Amyloodinium ocellatum* trophont. Note the peduncle with the rhizoid projections. Bar: 50 μm. (C) Lateral and ventral (D) view of *Trichodina* sp. (phase contrast). Bar: 10 μm.

The gills of all examined fish were found to be heavily infected with the dinoflagellate parasite *Amyloodinium ocellatum* and the ciliate parasite *Trichodina* sp. (Figure 1A and Video S1) while fewer parasites were on the skin. Various life stages of *A. ocellatum* , including trophonts, tomonts and dinospores, were observed attached to the gill surface. The trophonts exhibited the typical ovoidal shape, characterised by a peduncle with motile stomopode and rhizoids (Figure 1B). The average length of the trophonts was 97.9 μm (ranging from 71.1 to 131.7 μm). These trophonts were anchored in the fish gill epithelium using their rhizoids. At low‐power magnification, hundreds of trophonts could be seen attached to the gill lamellae in the viewing field of all observed fish. Similarly, numerous ciliate parasites, *Trichodina* sp. (Figure 1C,D), were also observed on the gills of the examined fish. These parasites were found attached to the gill epithelium and, in several instances, were even attached to the trophonts of *Amyloodinium ocellatum* (Figure 1A). The specimens exhibited a disc‐shaped body with a diameter ranging from 26.03 to 52.56 μm when viewed from the ventral side. The adhesive disc varied in diameter from 17.35 to 37.23 μm and was encircled by a finely striated border membrane, measuring 1.03 to 3.03 μm in width. The denticular ring had a diameter of 8.71 to 27.03 μm and featured 19 to 30 denticles.

**FIGURE 2:**
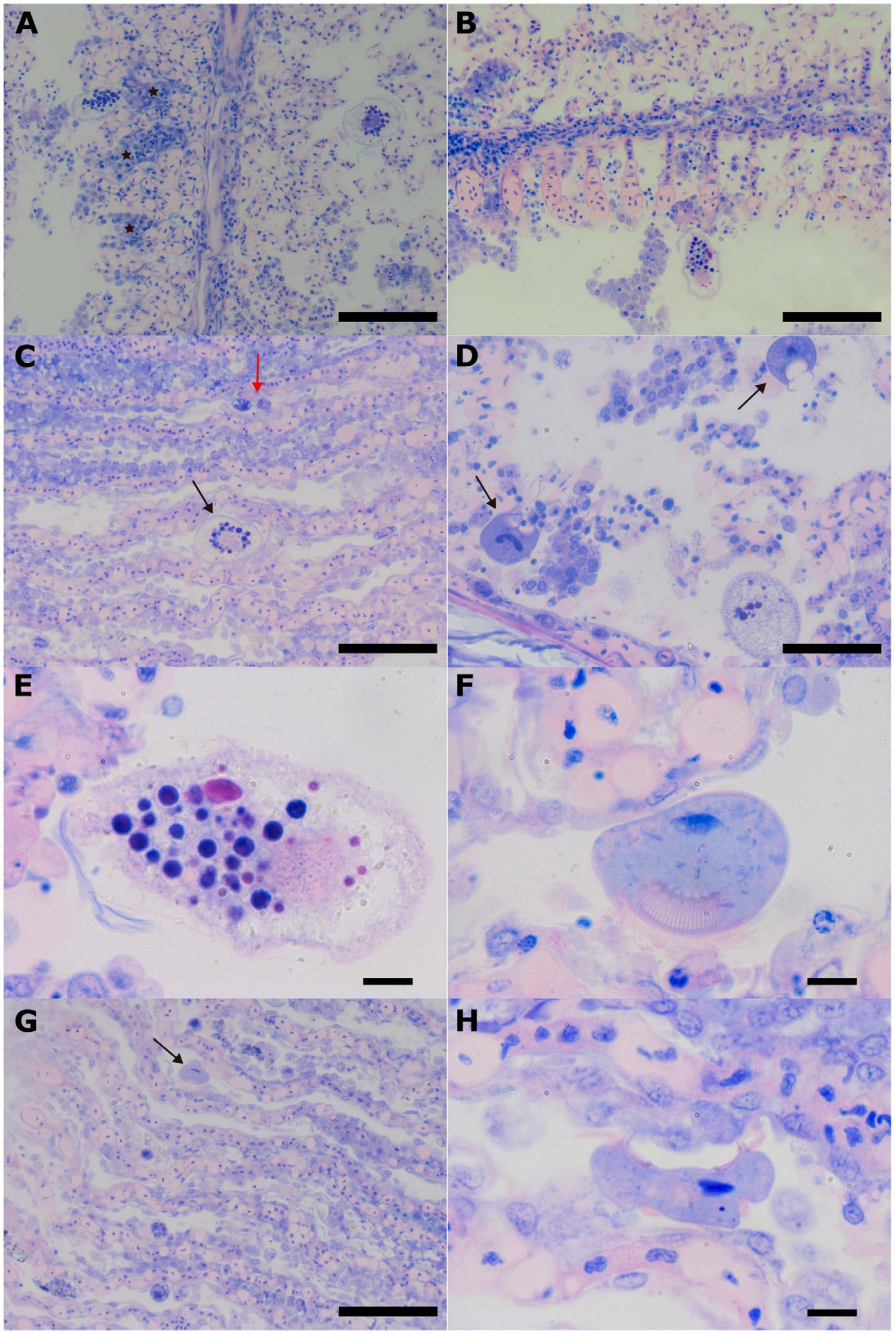
(A) Histological section of a flathead grey mullet gill showing two mature trophonts with translucent cytoplasm and basophilic granules. Multifocal hyperplasia and hypertrophy associated with the attachment of the parasite is indicated on the left (asterisks). (B) Leucocyte infiltration and dilation of the gill filament. (C) At the base of the gill lamellae, the epithelium is hyperplastic and hypertrophied, consisting of several cell layers. In these regions, the secondary lamellae are fused. Further along the lamellae, the epithelium ranges from cuboidal to flattened, with large vesicular nuclei and a moderate amount of eosinophilic cytoplasm. A few epithelial cells are necrotic, characterised by hypereosinophilia and either pyknotic or absent nuclei. The capillaries are diffusely congested. A mature trophont is marked with a black arrow, while two early trophonts are indicated with red arrows (D) Feeding activity of two *Trichodina* sp. (arrows) in a secondary lamellae area with numerous detached cells and cellular debris. In the lower right corner, an *Amyloodinium ocellatum* trophont is visible. (E) High magnification of the *Amyloodinium* trophont shown in panel B, revealing the basophilic granules and rhizoids. (F) High magnification of a *Trichodynia* specimen displaying the denticles and cilia, as well as bacteria internalised within its cytoplasm. (G) A trichodinid parasite attached on the gills of the secondary lamellae of infected fish (arrow). Early trophonts of *A. ocellatum* are also visible. (H) High magnification of a *Trichodina* sp. attached on the gill secondary lamellar epithelium. Scale bars: 100 μm in panels A–C and G; 50 μm in panel D; 10 μm in panels E, F and H.

The gills of the fish examined were severely affected by the parasitic infestation (Figure 2). Diffuse hydropic degeneration of the lamellar epithelium was the most common finding in all fish examined, often leading to necrosis of the epithelial cells. Additionally, hyperplasia and hypertrophy were predominantly observed at the bases of the secondary gill lamellae, frequently resulting in fusion (Figure 2B,C). Very few leukocytes such as macrophages and lymphocytes infiltrated the gill filaments (Figure 2B,D). *Amyloodinium* trophonts of varying sizes were observed in the gills (Figure 2C), with the largest trophonts exhibiting a more translucent cytoplasm containing vividly stained basophilic granules (Figure 2E). In contrast, smaller trophonts were more densely stained. The rhizoids were visible only in detached trophonts (Figure 2E). *Trichodina* sp. were less numerous in histological sections compared to fresh squash preparations, likely due to the fixation process. Nonetheless, many trichodinid parasites were observed between the gill lamellae, displaying feeding activity towards detached cells and cellular debris (Figure 2D), but also attached on the lamellar epithelium (Figure 2G,H). In several instances, bacteria were seen internalised within the parasites' cytoplasm (Figure 2F).

Dissolved oxygen levels were low, ranging from 1 to 3 mg/L, while the water temperature measured 30.4°C Most water quality parameters fell within acceptable ranges for sustaining fish, with the exception of BOD, which was critically high at 65 mg/L (File S2). The water was classified as brackish, with a salinity of 20 ppt.

## 4 Discussion

The mass mortality of flathead grey mullets in the Karteros River is attributed to severe gill damage caused by parasitic infestations from *Amyloodinium ocellatum* and *Trichodina* sp. The identification of *Amyloodinium ocellatum* at the species level was definitive due to its distinct morphological characteristics and the fact that the genus is monophyletic, containing only a single known species (Levy et al. 2007). In contrast, *Trichodina* sp. could not be identified to the species level due to the high diversity within the genus and the lack of sufficient molecular data. Both parasites have been associated with mass fish die‐offs on numerous occasions predominantly in aquaculture (Francis‐Floyd and Floyd 2011; Khan 2004; Mizuno et al. 2016; Paperna 1980; Saraiva et al. 2011), although there are some rare reports of mortalities in wild fish populations attributed to *Amyloodinium ocellatum* infestation (Kuperman and Matey 1999). *Amyloodinium ocellatum* is among the most virulent pathogens affecting fish, capable of causing up to 100% mortality in infected populations within a very short time frame. In aquaculture settings, fatalities often occur within 12–24 h of the parasite's detection (Moreira et al. 2023). This parasite has a worldwide distribution and has been reported in over 100 different fish hosts, including elasmobranchs. Pathological changes observed in the affected fish included hydropic degeneration of the gill epithelium, hyperplasia and hypertrophy, leading to lamellar fusion, along with focal infiltration of leukocytes and macrophages. Such host responses are commonly seen in various gill diseases, including parasitic infections caused by both parasites. For instance, hyperplasia, hypertrophy and lamellar fusion, along with degenerative and necrotic changes in the gill epithelium, were also observed in a study examining the pathology caused by *Trichodina puytoraci* in the same fish species, *Mugil cephalus* , in Egypt (Yemmen et al. 2011). Similarly, the same histopathological changes are observed in fish infected with *Amyloodinium ocellatum* (Paperna 1980), making it impossible to attribute the pathology to one parasite over the other. In the most comprehensive study of host–parasite interactions involving *Amyloodinium* in European seabass ( *Dicentrarchus labrax* ), it was demonstrated that lesion severity correlates with parasite load. The primary response was hyperplasia, accompanied by epithelial degeneration, edema, necrosis and limited leukocyte infiltration (Massimo et al. 2022). An interesting observation was the limited mucous present on their gills of examined fish. This was unexpected for the trichodinid parasites which are typically associated with excessive mucous production (Arafa Adly 2015) but could be explained by the concurrent infection by *Amyloodinium ocellatum*. According to Paperna (Paperna 1980), *Amyloodinium ocellatum* infections result in a significant reduction of mucous cells in the gills. This reduction could be attributed to the tissue's diminished capacity to regenerate mucous cells following prolonged infection, or it may represent a primary pathological feature of the disease.

While both parasites are commonly found on wild fish (Bahri 2012) and are distributed globally, including in Greece (Rigos and Katharios 2010), epizootic outbreaks in wild populations are rarely documented. To our knowledge, this report represents the first recorded instance of a fish kill attributed to these parasites. Mass mortalities from parasitic infections are less common in wild fish than in cultured populations, likely because natural ecosystems offer more dynamic conditions that help limit the spread and severity of infections. However, smaller‐scale outbreaks in wild populations may go undocumented or be difficult to trace. As previously mentioned, the river's delta had become disconnected from the sea, leading to the formation of a shallow, stagnant pond with water temperatures reaching 30°C–31°C. The water exhibited evident signs of eutrophication, characterised by algal growth, low oxygen levels and a significantly high biological oxygen demand (BOD), reflecting extremely poor water quality. While the elevated BOD may indicate organic pollution, it could also be attributed to the decomposition of dead fish, which were already decaying at the time of sampling. These conditions are ideal for parasite proliferation, while simultaneously pushing the fish to their physiological limits, thereby compromising their immunocompetence. A similar incident occurred in August 2019 at the Aposelemis Dam, located approximately 25 km west of the Karteros River. In this case, goldfish ( *Carassius auratus* ) trapped in a side stream of the Aposelemis reservoir succumbed to a mass infestation of *Ichthyobodo necator*, a flagellate protozoan gill parasite. The outbreak was exacerbated by minimal water exchange and elevated water temperatures. During the summer months, Crete experiences prolonged droughts, which severely reduce surface water volumes. This leads to environmental stress on fish populations, including low oxygen levels, high water temperatures and an increased risk of disease outbreaks.

The Karteros River delta is designated as a small natural island wetland and, as such, is a strictly protected area under a Presidential Decree issued by the Greek State in 2012. However, despite its legal protection, the region suffers from the effects of uncontrolled urbanisation and intensive agricultural activities (Chatzidavid et al. 2023; Kourgialas et al. 2024). Additionally, the river's upstream area borders the industrial zone of Heraklion, compounding the pressures on water quality. These factors could collectively contribute to the ongoing degradation of the river's ecosystem. Immediate action is needed to safeguard this area, one of the few remaining natural wetlands on Crete.

## Author Contributions

**Pantelis Katharios:** conceptualization, methodology, writing – original draft, writing – review and editing, resources, supervision, formal analysis, validation, investigation. **Evangelia Karageorgou:** investigation, writing – review and editing, formal analysis.

## Conflicts of Interest

The authors declare no conflicts of interest.

## Supporting information


**Video S1.** Video showcasing a fresh preparation of gill samples from infected fish under a microscope, highlighting the presence of parasites and the severity of the infection.

**File S1.** Satellite picture of Karteros River and picture from the banks of the river during the incident.

**File S2.** Physicochemical parameters of water of Karteros River.

## Data Availability

The data that support the findings of this study are available from the corresponding author upon reasonable request.
