# Supplementary material for: Protozoan Co‐Infection Drives Fish Mortality Event in Crete's Karteros River
Source: J Fish Dis. 2025 Feb 24;48(6):e14099. doi: 10.1111/jfd.14099 (PMC12068841; doi:10.1111/jfd.14099)
Supplement: Supplementary file 2 — File S1. Satellite picture of Karteros River and picture from the banks of the river during the incident. [file JFD-48-e14099-s001.docx]

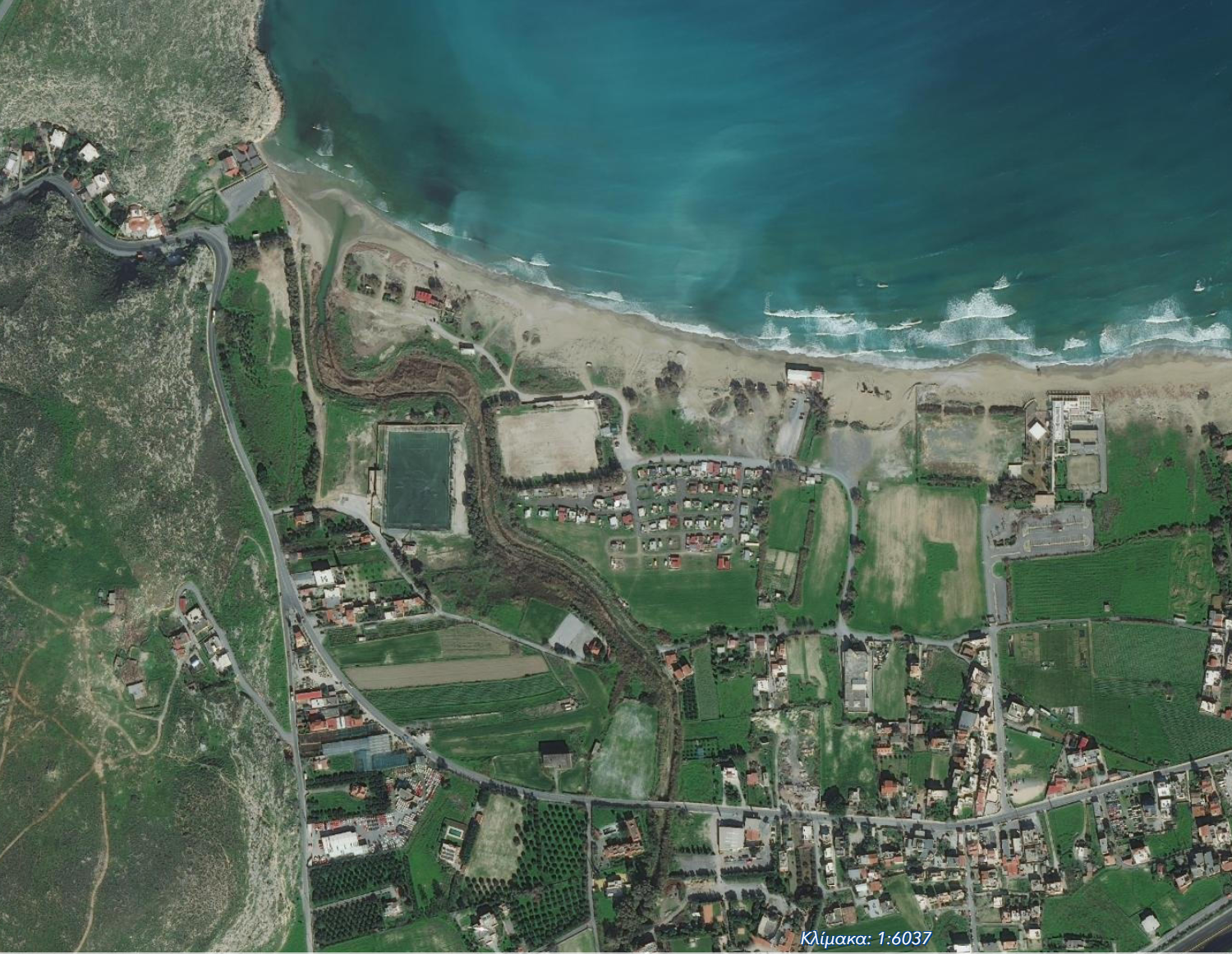


Satellite photograph of the Karteros River delta (red star) where the incidence was recorded


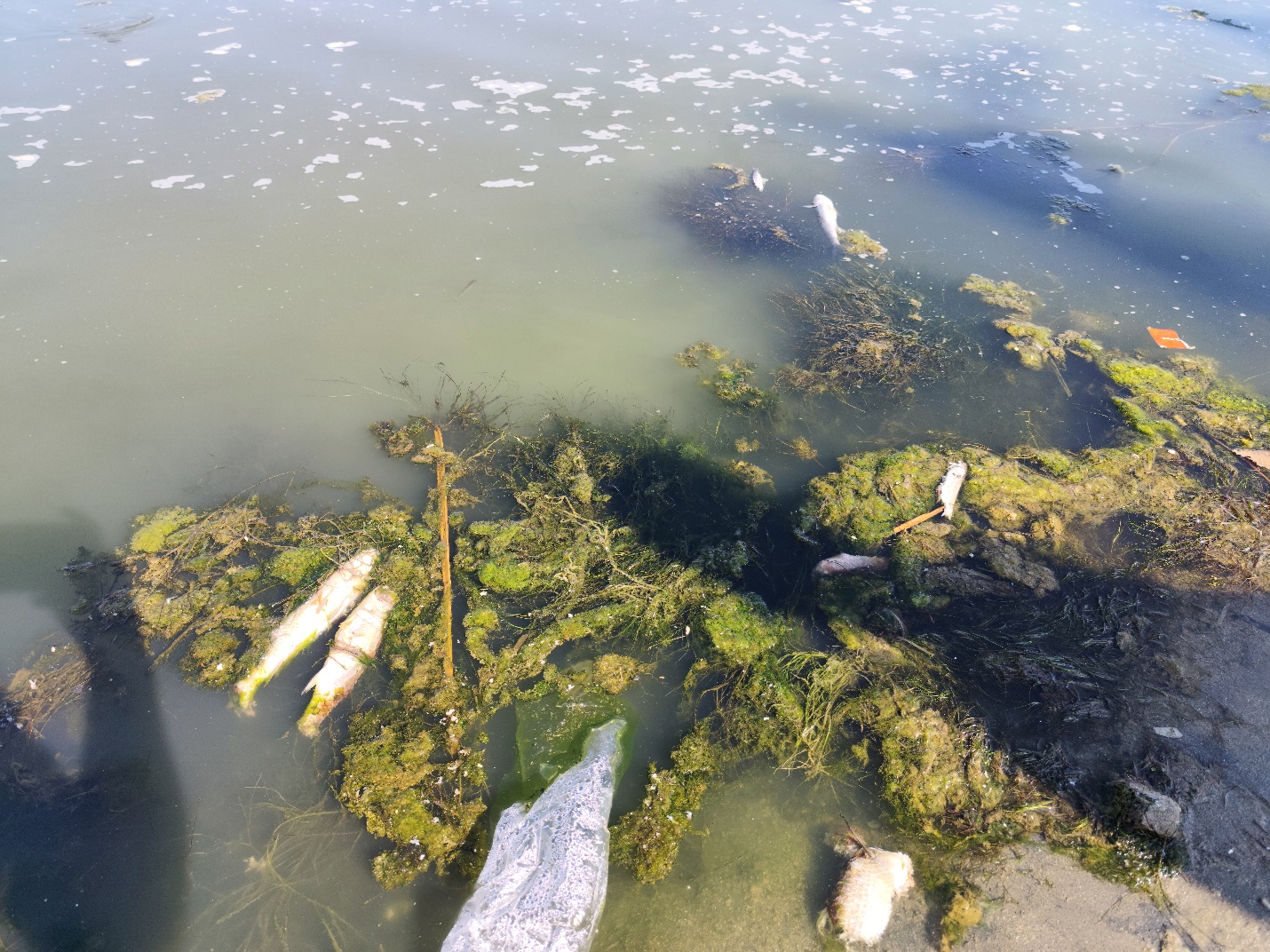


Dead fish along the riverbank, surrounded by visible algal growth, illustrating the effects of eutrophication.
